# On the Organization of Hospitals for the Insane

**Published:** 1854-04-01

**Authors:** 


### ON THE ORGANIZATION OF HOSPITALS FOR THE INSANE.

I. The general controlling power should be vested in a Board of Trustees, or Managers; if of a State institution, selected in such manner as will be likely most effectually to protect it from all influences connected with political measures or political changes, if of a private corporation, by those properly authorized to vote.

II. The Board of Trustees should not exceed twelve in number, and be composed of individuals possessing the public confidence, distinguished for liberality, intelligence, and active benevolence; above all political influence, and able and willing faithfully to attend to the duties of their station. Their tenure of office should be so arranged, that when changes are deemed desirable, the terms of not more than one-third of the whole number should expire in any one year.

III. The Board of Trustees should appoint the Physician, and on his nomination, and not otherwise, the Assistant Physician, Steward, and Matron. They should as a board, or by committee, visit and examine every part of the institution, at frequent stated intervals, not less than semi-monthly, and at such other times as they may deem expedient, and exercise so careful a supervision over the expenditure and general operations of the Hospital, as to give to the community a proper degree of confidence in the correctness of its management.

IV. The Physician should be the Superintendent and chief executive officer of the establishment. Besides being a well-educated physician, he should possess the mental, physical, and social qualities to fit him for the post. He should serve during good behaviour, reside on, or very near the premises, and his compensation should be so liberal, as to enable him to devote his whole time and energies to the welfare of the hospital. He should nominate to the Board suitable persons to act as Assistant Physician, Steward, and Matron. He should have entire control of the medical, moral, and dietetic treatment of the patients, the unrestricted power of appointment and discharge of all persons engaged in their care, and should exercise a general supervision and direction of every department of the Institution.

V. The Assistant Physician, or Assistant Physicians, where more than one are required, should be graduates of medicine, of such character and qualifications as to be able to represent and to perform the ordinary duties of the Physician during his absence.

VI. The Steward, under the direction of the Superintending Physician, and by his order, should make all purchases for the institution, keep the accounts, make engagements with, pay, and discharge those employed about the establishment; have a supervision of the farm, garden, and grounds, and perform such other duties as may be assigned him.

VII. The Matron, under the direction of the Superintendent, should have a general supervision of the domestic arrangements of the house; and under the same direction, do what she can to promote the comfort and restoration of the patients.

VIII. In institutions containing more than two hundred patients, a Second Assistant Physician and an Apothecary should be employed; to the latter of whom other duties, in the male wards, may be conveniently assigned.

IX. If a Chaplain is deemed desirable as a permanent officer, he should be selected by the Superintendent, and, like all others engaged in the care of the patients, should be entirely under his direction.

X. In every Hospital for the Insane, there should be one Supervisor for each sex, exercising a general oversight of all the attendants and patients, and forming a medium of communication between them and the officers.

XI. In no institution should the number of persons in immediate attendance on the patients be in a lower ratio than one attendant for every ten patients; and a much larger proportion of attendants will commonly be desirable.

XII. The fullest authority should be given to the Superintendent to take every precaution that can guard against fire or accident within an institution, and to secure this an efficient night-watch should always be provided.

XIII. The situation and circumstances of different institutions may require a considerable number of persons to be employed in various other positions; but in every hospital, at least all those that have been referred to are deemed not only desirable, but absolutely necessary, to give all the advantages that may be hoped for from a liberal and enlightened treatment of the insane.

XIV. All persons employed in the care of the insane should be active, vigilant, cheerful, and in good health. They should be of a kind and benevolent disposition; be educated, and in all respects trustworthy; and their compensation should be sufficiently liberal to secure the services of individuals of this description.

